# A mutualistic model bacterium is lethal to non-symbiotic hosts via the type VI secretion system

**DOI:** 10.1128/mbio.00157-25

**Published:** 2025-04-17

**Authors:** Keegan E. Gaddy, Alecia N. Septer, Karen Mruk, Morgan E. Milton

**Affiliations:** 1 Department of Biochemistry and Molecular Biology, Brody School of Medicine, East Carolina University 3627, Greenville, North Carolina, USA; 2 Department of Earth, Marine and Environmental Sciences, University of North Carolina at Chapel Hill, Chapel Hill, North Carolina, USA; 3 Department of Pharmacology and Toxicology, Brody School of Medicine, East Carolina University 3627, Greenville, North Carolina, USA; University of Connecticut, Storrs, Connecticut, USA

**Keywords:** pathogenicity, *Vibrio fischeri*, zebrafish, type IV secretion system

## Abstract

What makes a bacterium pathogenic? Since the early days of germ theory, researchers have categorized bacteria as pathogens or non-pathogens, those that cause harm and those that do not, but this binary view is not always accurate. *Vibrio fischeri* is an exclusive mutualistic symbiont found within the light organs of Hawaiian bobtail squid. This symbiotic interaction requires *V. fischeri* to utilize a range of behaviors and produce molecules that are often associated with pathogenicity. This juxtaposition of employing “pathogenic” behaviors for a symbiotic relationship led the field to focus on how *V. fischeri* establishes a beneficial association with its host. In this study, we observe that *V. fischeri* induces mortality in zebrafish embryos and *Artemia* nauplii. Non-lethal doses of *V. fischeri* lead to zebrafish growth delays and phenotypes indicative of disease. Our data also provide evidence that the conserved type VI secretion system on chromosome I (T6SS1) plays a role in the *V. fischeri*-induced mortality of zebrafish embryos and *Artemia* nauplii. These results support the hypothesis that the *V. fischeri* T6SS1 is involved in eukaryotic cell interactions. Despite its traditional view as a beneficial symbiont, we provide evidence that *V. fischeri* is capable of harming aquatic organisms, indicating its potential to be pathogenic toward non-symbiotic hosts.

**IMPORTANCE:**

*Vibrio fischeri* is best known for its beneficial partnership with the Hawaiian bobtail squid, where it uses molecular tools often associated with disease-causing bacteria. Our research shows that *V. fischeri* can also cause harm, killing zebrafish embryos and brine shrimp larvae. We pinpoint one of *V. fischeri*’s two type VI secretion systems (T6SS1) as a key factor in this pathogenicity. These findings reveal that *V. fischeri* is not strictly a mutualistic microbe but can act like a pathogen under certain conditions. This broadens our understanding of how *V. fischeri* could interact with different hosts and offers new insights into the dual roles bacteria can play in nature.

## OBSERVATION

The *Vibrio* genus encompasses diverse marine bacteria found globally with species exhibiting free-living, symbiotic, or pathogenic lifestyles (1). As a powerful model organism for bacteria-host interactions, *Aliivibrio fischeri* (referred to here as *Vibrio fischeri* for consistency with previous literature) has been extensively studied for its mutualistic relationship with the Hawaiian bobtail squid, *Euprymna scolopes* (2–4). Interestingly, many of the *V. fischeri* processes involved in symbiosis parallel pathogen-host interactions (2). During symbiosis establishment, *V. fischeri* releases lipopolysaccharides, peptidoglycan monomers, and small RNAs (2, 5, 6) to direct host development, form biofilm, and evade immune cells (2). As such, *V. fischeri* challenges the conventional view of pathogenicity by employing “pathogenic” mechanisms for beneficial symbiosis. Yet a critical question remains: If *V. fischeri* is equipped with the tools of a pathogen, what prevents it from exhibiting harmful behavior?

Fish and shrimp are animals that *V. fischeri* encounter in its natural habitat as it transitions between symbiotic hosts. Zebrafish (*Danio rerio*) are an ideal model organism for studying bacterial infections and host immune response and are used to explore the pathogenesis and virulence of disease-causing *Vibrio* (7). Moreover, *Artemia* nauplii are used as an aquatic host to test pathogenicity of *Vibrio parahaemolyticus* and *Vibrio coralliiliticus* (8, 9). Therefore, we used zebrafish embryos and *Artemia* nauplii to address the question of potential *V. fischeri* pathogenicity. Since the T6SS is an important virulence factor for many pathogenic bacteria (10, 11), we tested the conserved T6SS on *V. fischeri* chromosome I (T6SS1) for its potential role in pathogenicity. To date, no role has been linked to the T6SS1, but it is hypothesized to be involved in eukaryotic cell interaction (12). Here, we provide evidence that directly supports this hypothesis. Our results set a new precedent by indicating there are conditions under which *V. fischeri* acts as a pathogen, expanding the field into the realm of pathogenicity.

**Fig 1:**
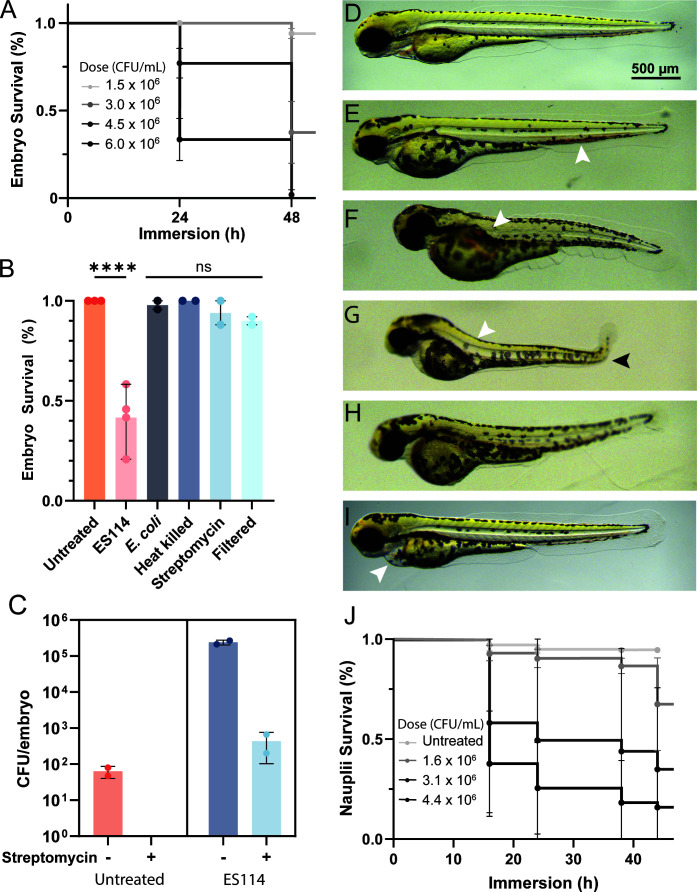
*V. fischeri* is lethal to zebrafish embryos and *Artemia* nauplii. (**A**) Survival curves of zebrafish embryos immersed with ES114 (*n* = 48 across 2 replicates). (**B**) Survival is dependent on viable *V. fischeri* (*n* = 32 across, 2 replicates). (**C**) Quantification of bacteria from zebrafish embryos with and without streptomycin treatment. Each point represents independent replicates of eight embryos. (**D–I**) Representative micrographs of zebrafish phenotypes in control (**D**) and embryos exposed to *V. fischeri* for 48 h (**E–I**). Arrowheads indicate blood pooling, morphological defects in the tail, necrotic tissue, and pericardial edema. (**J**) Survival curve of *Artemia* nauplii immersed with *V. fischeri* ES213 over 44 h (*n* ≥ 66 across 2 replicates). Bar charts represent mean ± SD. ns, nonsignificant; ****P < 0.0001.

The impact of *V. fischeri* ES114 exposure on zebrafish embryos in their chorions was tested using a bath immersion infection model. Zebrafish mortality was dose-dependent and increased with immersion time (Fig. 1A). To determine whether embryo mortality is caused by direct effects of *V. fischeri* exposure, several control scenarios were tested. Zebrafish immersed in equivalent concentrations of *Escherichia coli* all survived (Fig. 1B), suggesting mortality is not generally due to the presence of bacteria. We confirmed zebrafish survival can be recovered by treating *V. fischeri* with streptomycin or heat-killing prior to exposure (Fig. 1B), indicating live cells are required for mortality. Finally, to test whether mortality was caused by the accumulation of extracellular compounds, E3 media incubated with *V. fischeri* overnight was filter-sterilized before embryo immersion. Survival rates were similar to untreated embryos (Fig. 1B), suggesting *V. fischeri* is not releasing embryo-lethal compounds into the media. These results establish that the zebrafish mortality is dependent on viable *V. fischeri* interacting with the embryos. Zebrafish chorions provide a protective layer for the embryo while still allowing the exchange of oxygen and ions through regularly spaced pores. The outer pore channel opening is 0.5–0.7 µm in diameter (13). This is theoretically wide enough for *V. fischeri,* average 0.6 ± 0.1 in diameter (14), to pass through. This suggests that while embryos were exposed to *V. fischeri* in their chorions, the bacteria can still access the embryo.

Because mortality required live cells and spent supernatant was not toxic to embryos, we predicted that *V. fischeri* may be in direct contact with embryos. We evaluated *V. fischeri* associated with fish tissue by quantifying colony-forming units (CFUs). Washed and homogenized embryos with chorions removed had ~2.4 × 10^5^ CFU per embryo (Fig. 1C). Embryos treated with an additional 30 min streptomycin wash prior to homogenization had ~4.3 × 10^2^ CFU per embryo (Fig. 1C). This indicates while many bacteria are localized to the embryo surface, a portion may be localized within the tissue.

Embryos that survive *V. fischeri* exposure presented with developmental defects. The most common morphological changes are shorter tail length, smaller head, necrotic tissue, and abnormal tail curvature (Fig. 1D through I). Less common changes included pericardial edema (Fig. 1I). After 38 h of immersion, the most typical indicator of embryo death was slowing of the heart rate and total cessation of blood flow throughout the trunk and tail. At 48 h of immersion, most embryos displaying diminished blood flow had no heartbeat and began coagulation of the tail and yolk sac (Fig. 1F and H). Embryos that did not have blood flow, but still had a noticeable heartbeat, show sporadic pectoral fin movement in response to disturbance but were incapable of large movements. These progressive morphological impairments underscore the severe impact of *V. fischeri* on zebrafish embryonic development, indicating the nature of *V. fischeri* on animal development is host-specific and can range from beneficial to pathogenic (15). Exposure of *Artemia* nauplii to *V. fischeri* ES114 revealed a similar dose-dependent lethality (Fig. 1J). Taken together, the data indicate that *V. fischeri* can act as a pathogen to multiple aquatic species.

**Fig 2:**
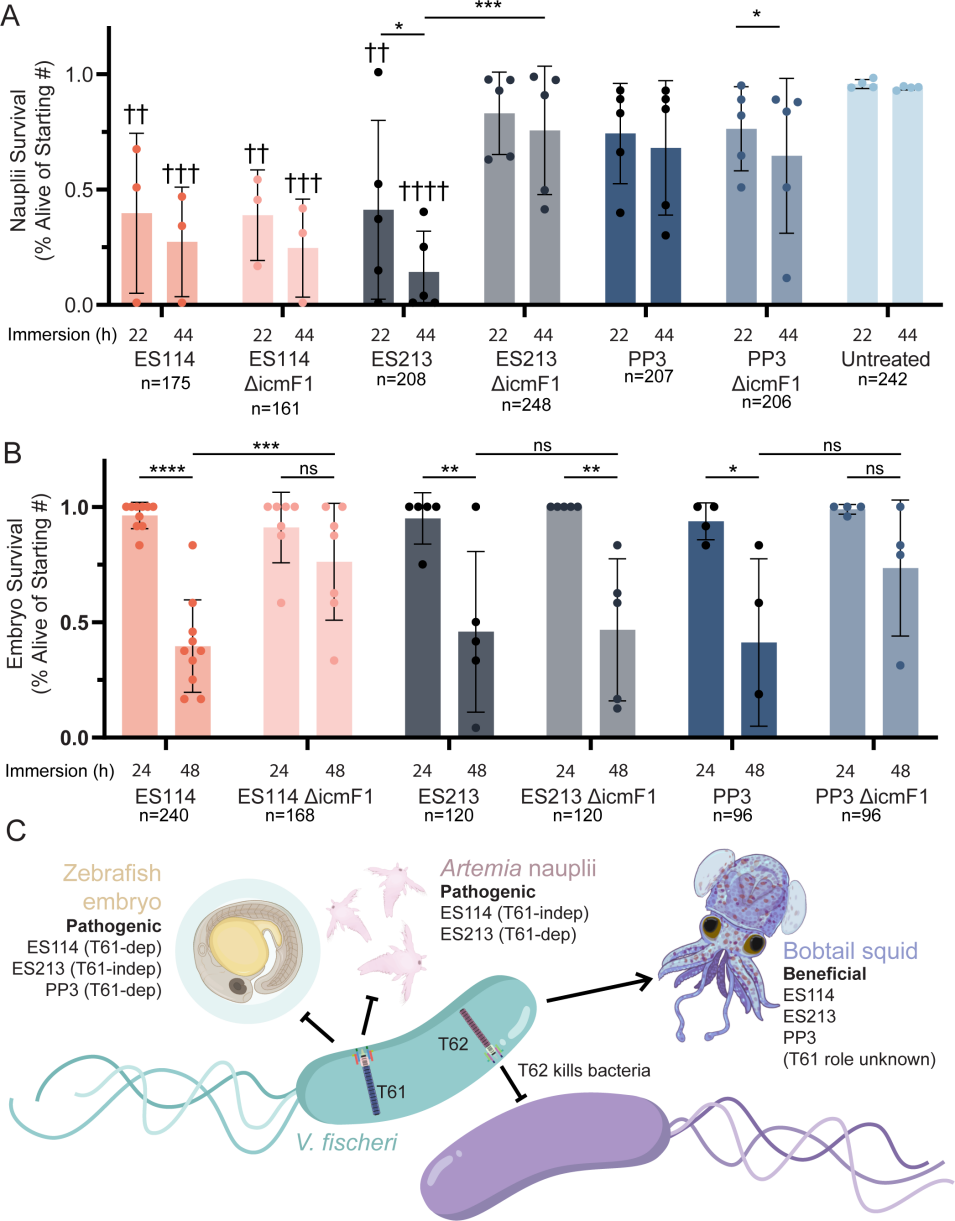
T6SS1 impacts embryo mortality. (**A**) Survival of *Artemia* nauplii immersed in 4.6 × 10^6^ CFU/mL *V*. *fischeri* across three replicates. (**B**) Survival of zebrafish embryos immersed in 3.0 × 10^6^ CFU/mL *V*. *fischeri* across four replicates. Untreated control all survived. Bar charts represent mean ± SD. Asterisks show significance between WT and mutant pairs, and daggers show significance with untreated controls. ns, nonsignificant; *, *P* < 0.05; ** and ††, *P* < 0.01; *** and †††, *P* < 0.001; **** and ††††, *P* < 0.0001. (**C**) Model for *V. fischeri* effects on host health and the role of T6SS1, made with the help of Biorender.com.

Our discovery that *V. fischeri* can exhibit pathogenic behavior raises a crucial question: What virulence factors drive lethality? Given the critical role of the T6SS in pathogenicity (16), we investigated whether T6SS contributes to *V. fischeri* pathogenic effects. The T6SS is a molecular syringe used to deliver payloads of effectors with diverse functions. *V. fischeri* has two T6SS loci (T6SS1 and T6SS2). T6SS2 functions as an antibacterial weapon used to establish mono-colonized crypts in *E. scolopes* light organs (17). While T6SS2 is encoded by roughly half of sequenced strains, T6SS1 is conserved in all isolates, but with an unknown function (12). The T6SS1 locus in strain ES114 is not essential for squid colonization (18). To determine the extent to which *V. fischeri* lethality is conserved across strains and the role of T6SS1, three symbiotic *V. fischeri* strains (ES114, ES213, and PP3) containing a deletion of the T6SS1 *icmF* gene, which encodes an essential structural protein, were compared to their wild-type (WT) parent in *Artemia* nauplii (Fig. 2A) and zebrafish embryo (Fig. 2B) survival studies.

Deletion of *icmF1* resulted in a host- and strain-specific increase in survival. Nauplii survival remained low for animals exposed to ES114 ∆*icmF1*, suggesting T6SS1 is dispensable for nauplii lethality in this strain. Nauplii showed similar levels of survival for PP3 WT or PP3 ∆*icmF1* that were not statistically different from the no-exposure control, suggesting PP3 lethality for nauplii is low. However, ES213 WT exposure resulted in low nauplii survival that was significantly higher when *icmF1* was deleted, suggesting a role for T6SS1 in ES213 lethality against *Artemia*.

Interestingly, the strain-specific lethality phenotypes were reversed for zebrafish embryos (Fig. 2B). ES213 ∆*icmF1* retained lethality, similar to the WT parent, suggesting T6SS1 is dispensable for this strain. However, embryos exposed to the *icmF1* mutants for both ES114 and PP3 showed higher survival rates when compared to their WT parents. Although the difference between zebrafish survival for PP3 WT and PP3 ∆*icmF1* was not statistically significant, the effect size is consistent, and the *icmF1* mutant exposed embryos reached higher levels of survivability, compared to WT, for each of the four independent trials. These results suggest that, under our experimental conditions, the T6SS1 is involved in pathogenesis and supports the hypothesis that T6SS1 mediates interactions with eukaryotic hosts. This conclusion is supported by the fact that the different T6SSs in *V. coralliilyticus* have distinct roles. One is involved in inter-bacterial competition similar to the *V. fischeri* T6SS2, while the other is directly involved in targeting the eukaryotic host (8).

In summary, *V. fischeri’s* pathogenic behavior toward non-symbiotic hosts expands its utility as a model organism beyond that of beneficial symbiosis (Fig. 2C). Our observations raise a series of unanswered questions including What conditions trigger *V. fischeri* pathogenicity? Are there additional *V. fischeri* virulence factors involved in this process? And will the development of the zebrafish immune system reduce the pathogenic behavior? Future work will employ this multi-strain and multi-host model system to explore mechanistic connections between beneficial and harmful infection.

## SUPPLEMENTAL MATERIAL

Supplemental Material mbio.00157-25-s0001.pdf Supplemental materials and methods and Table S1.

ASM does not own the copyrights to Supplemental Material that may be linked to, or accessed through, an article. The authors have granted ASM a non-exclusive, world-wide license to publish the Supplemental Material files. Please contact the corresponding author directly for reuse.
